# Glucagon-like peptide-1 (GLP-1) receptor agonists in inflammatory bowel disease: mechanisms, clinical implications, and therapeutic potential

**DOI:** 10.1093/ecco-jcc/jjaf167

**Published:** 2025-09-16

**Authors:** Michael Colwill, Sebastian Povlsen, Richard Pollok, Kamal Patel, James Goodhand, Tariq Ahmad, Sailish Honap

**Affiliations:** Department of Gastroenterology, St George’s University Hospital NHS Foundation Trust, London, United Kingdom; Institute for Infection and Immunity, City St George’s University Medical School, London, United Kingdom; Department of Gastroenterology, Guy’s and St Thomas’ NHS Foundation Trust, London, United Kingdom; Institute for Infection and Immunity, City St George’s University Medical School, London, United Kingdom; Department of Gastroenterology, St George’s University Hospital NHS Foundation Trust, London, United Kingdom; Department of Gastroenterology, Royal Devon and Exeter Hospital, Exeter, United Kingdom; Department of Gastroenterology, Royal Devon and Exeter Hospital, Exeter, United Kingdom; Department of Gastroenterology, St George’s University Hospital NHS Foundation Trust, London, United Kingdom; School of Immunology and Microbial Sciences, King’s College London, London, United Kingdom

**Keywords:** glucagon-like peptide-1 receptor agonists, inflammatory bowel disease, Crohn’s disease, ulcerative colitis

## Abstract

Glucagon-like peptide-1 receptor agonists are increasingly recognized for their potential dual benefit in inflammatory bowel disease (IBD), offering metabolic advantages alongside emerging anti-inflammatory, immunomodulatory, and gut barrier-enhancing effects. Pre-clinical data demonstrate attenuation of inflammation, preservation of epithelial integrity, and modulation of the microbiome in colitis models. Early retrospective studies in patients with IBD suggest improved clinical outcomes, such as reduced hospitalization and surgery rates, particularly in those with obesity. Glucagon-like peptide-1 receptor agonists are already widely used for obesity and diabetes, including increasing self-administration by patients outside medical supervision. Their impact on drug absorption, safety in gastrointestinal disease, and interactions with existing IBD therapies require further exploration. This review synthesizes the mechanistic rationale, pre-clinical evidence, and clinical data to date, highlighting the potential utility and safety considerations of glucagon-like peptide-1 receptor agonists in IBD and emphasizes the need for robust prospective trials to ascertain their safety and efficacy in this patient population.

## 1. Introduction

The incidence and prevalence of both inflammatory bowel disease^1^ (IBD) and obesity are rising globally with projections suggesting 3.8 billion people could be overweight or obese by 2050.^2^ This pandemic of obesity has placed a substantial burden on healthcare systems and patients alike, sparking intense interest in the development of novel therapeutic strategies. Glucagon-like peptide-1 receptor agonists (GLP-1RAs) are a class of agents where use has surged in recent years, with global sales projected to reach $100 billion by 2030.^3^ Originally developed for glycemic control and weight loss, GLP-1RAs are now attracting attention for their potential anti-inflammatory, immunomodulatory, and gut barrier-enhancing properties—making them a promising therapeutic candidate in the context of IBD.

GLP-1RAs, approved for type 2 diabetes (T2DM), enhance incretin signaling to improve postprandial glucose metabolism.^4^ Additional effects include delayed gastric emptying, reduced gastric acid secretion, and appetite suppression via hypothalamic pathways, leading to significant weight loss in large randomized controlled trials (RCTs).^5^^,^^6^ Beyond metabolic effects, GLP-1RAs may reduce cardiovascular risk through modulation of inflammation and endothelial function.^7^ In the gut, GLP-1RAs enhance epithelial barrier integrity and downregulate pro-inflammatory cytokines.^8^^,^^9^ Relatedly, the GLP-2RA teduglutide, licensed for short bowel syndrome, promotes mucosal healing and intestinal growth.^10^ Together, these findings suggest GLP-1RAs may offer a dual-action therapeutic approach in IBD by targeting both metabolic dysfunction and intestinal inflammation.

Despite these mechanistic insights, our clinical understanding of GLP-1RAs in patients with IBD remains limited. The body mass index (BMI) of individuals with IBD has seen an upward shift with 15%-40% of adult patients reported to be obese and 20%-40% overweight.^11^ Obesity contributes to an inflammatory state through adipokines and various pro-inflammatory cytokines^12^ and is associated with higher risks of having active disease,^12^ poorer surgical outcomes,^13^ and impaired quality of life in IBD.^14^ Obesity can also impact the pharmacokinetics of therapies through changes to volume of distribution and drug clearance,^15^ and may negatively alter the efficacy of therapies used to treat IBD.^16^^,^^17^ Given this, the weight loss and metabolic improvements associated with GLP-1RA therapy may provide tangible benefits for this population. However, several unanswered questions remain—namely, whether GLP-1RAs, given their common gastrointestinal side-effects such as nausea, vomiting, and altered bowel habits, are safe and tolerable in patients with IBD; whether they might serve as adjunctive therapies in this context; and whether any pharmacological interactions exist between GLP-1RAs and conventional IBD treatments. These knowledge gaps pose barriers to translation and have contributed to the absence of clinical guidance for their use in this setting.

In light of increasing GLP-1RA use and these significant knowledge gaps, we conducted a structured literature search in March 2024 using the terms “GLP-1 receptor agonists” AND “inflammatory bowel disease” OR “ulcerative colitis” OR “Crohn’s disease.” This narrative review is, to our knowledge, the first to comprehensively explore the pathophysiological rationale, pre-clinical evidence, and clinical considerations of GLP-1RA use in IBD.

## 2. GLP-1 discovery, physiology, and therapeutic targeting

GLP-1 is a peptide hormone produced by the cleavage of proglucagon.^18^ It was first discovered in the 1980s by Lund et al., who sequenced recombinant plasmids containing cDNA encoding pre-proglucagon from the anglerfish. This revealed that the gene encoded not only glucagon but also an additional glucagon-like peptide.^19^ Subsequent studies confirmed two distinct glucagon-like peptides, which were later termed GLP-1 and GLP-2.^20–23^ The biological activity of these peptides was first demonstrated in 1987 through its insulinotropic effects for GLP-1,^24^ and later in 1996 for GLP-2.^25^^,^^26^

**Figure 1.**
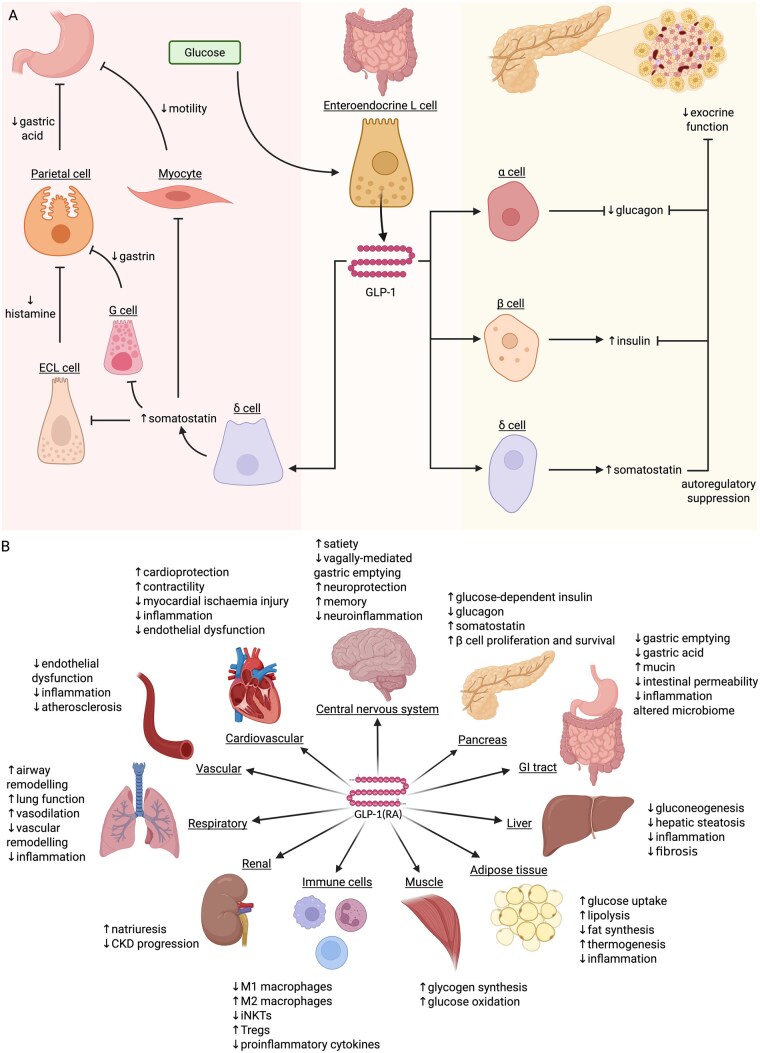
(A) Classical GLP-1 signaling in the pancreas and stomach. (B) Multi-organ effects of GLP-1 signaling. Figures created in BioRender. Povlsen, S. (2025), https://BioRender.com/kygr09l

Enteroendocrine L-cells of the small and large intestine produce both GLP-1 and GLP-2 in response to luminal glucose, with both hormones acting through G protein-coupled receptors distributed across multiple organ systems (Figure 1).^27^ In the pancreas, GLP-1 receptor (GLP-1R) activation in islet cells promotes glucose-dependent insulin secretion from β-cells,^28^ inhibits glucagon release from α-cells, and suppresses exocrine function via induction of pancreatic somatostatin. In the stomach, stimulation of gastric δ-cells leads to somatostatin release, resulting in reduced gastric acid secretion and delayed gastric emptying (Figure 1A).^18^^,^^29^ GLP-1 also reduces bile acid synthesis, release from the gallbladder, and ileal reabsorption.^30^ GLP-1Rs are expressed in Brunner’s glands, parietal and smooth muscle cells of the stomach, neurons of the myenteric plexus, and enteroendocrine cells. Expression extends to intraepithelial lymphocytes (IELs), particularly in the duodenum, ileum, and colon—though sparing of the cecum and distal colon has been suggested.^31^ Receptor expression is also seen in immune populations, including thymocytes, splenocytes, bone marrow-derived cells, and peripheral regulatory T cells.^32^ Lastly, in the context of gastrointestinal physiology, GLP-1 is also produced by neurons in the nucleus of the solitary tract in the brainstem.^27^ It delays gastric emptying via vagally mediated parasympathetic activity,^33–35^ and regulates satiety through hypothalamic and mesolimbic circuits.^36^

Endogenous GLP-1 has a short half-life (1-2 min), with only ∼10% reaching systemic circulation due to rapid degradation, which has driven the development of degradation-resistant GLP-1RAs.^37^ Exenatide, approved in 2005 following its 1992 discovery, was followed by longer-acting agents including liraglutide, albiglutide, dulaglutide, lixisenatide, and semaglutide. Tirzepatide, a dual glucose-dependent insulinotropic polypeptide (GIP)/GLP-1RA, has recently been licensed, and retatrutide—a triple GIP/GLP-1/glucagon receptor agonist—shows promising weight loss effects in ongoing phase 3 trials.^38–40^ Orally bioavailable small-molecule GLP-1RAs are in early development, offering potential for once-daily administration.^41–44^ Early data from the ACHIEVE clinical trial program announced by Lilly demonstrate efficacy of orforglipron, an oral GLP-1RA, without additional safety concerns, and the full trial results are awaited.^45^

The systemic distribution of GLP-1Rs underpins the diverse actions of GLP-1RAs beyond glycemic control and weight loss (Figure 1B).^32^^,^^46–49^ This has stimulated interest in their potential in other chronic diseases, particularly those with inflammatory components.^27^ Given their anti-inflammatory effects and intestinal receptor expression, GLP-1RAs may offer a new avenue for intervention in IBD.

## 3. GLP-1RAs in inflammatory bowel disease: preclinical evidence

### 3.1. Immune modulation

The evidence for GLP-1 signaling in immune function stems from studying the physiological response to inflammation, where GLP-1 release increases in rodent inflammation models and in humans with systemic inflammation, where it correlates with interleukin (IL)-6 levels, and in a murine anti-CD3 model of intestinal inflammation, where GLP-1R expression is upregulated.^50–52^

**Figure 2.**
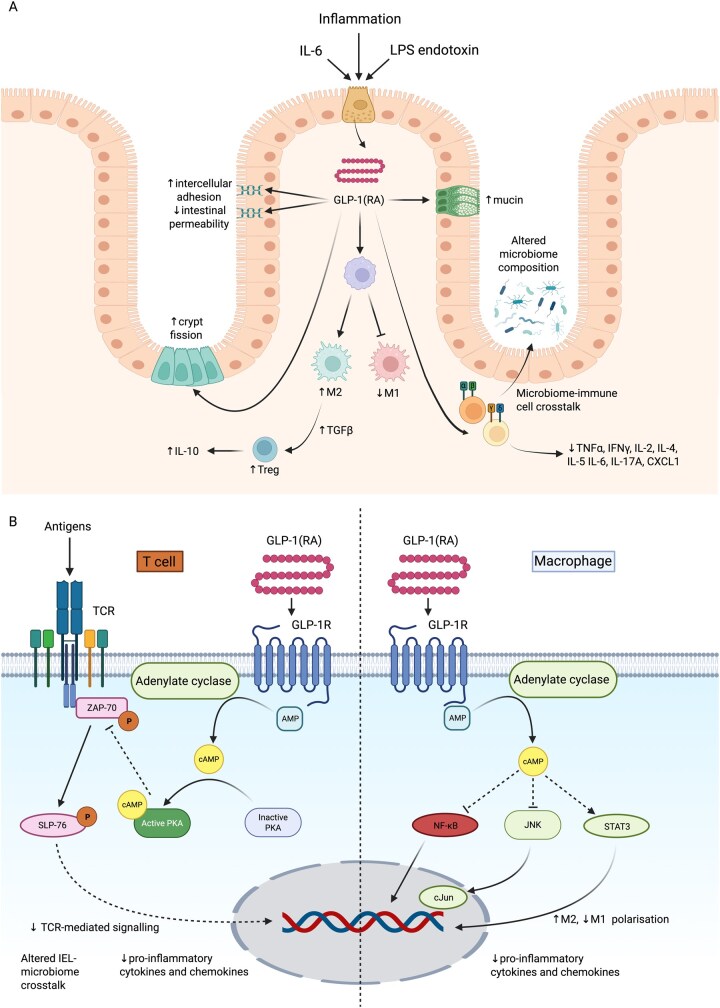
(A) Inflammatory stimuli such as interleukin-6 (IL-6) and lipopolysaccharide (LPS) endotoxin trigger secretion of GLP-1 by enteroendocrine L cells in the intestine. GLP-1 signaling, via endogenous GLP-1 or GLP-1RAs, induces M2 macrophage polarization and reduces M1 polarization. M2 macrophages, in turn, promote regulatory T cell (Treg) differentiation through TGF-β signaling and support IL-10 production, further amplifying anti-inflammatory effects. GLP-1 also acts directly on αβ and γδ T cells, reducing pro-inflammatory cytokine and chemokine secretion, altering microbiome–immune cell interactions, and modulating gut microbial composition. Additionally, GLP-1 signaling exerts intestinotrophic effects by stimulating crypt fission, enhancing epithelial barrier integrity through increased intercellular adhesion, and promoting mucin production. (B) Intracellular GLP-1 signaling mechanisms in immune cells. T cell receptor (TCR)-mediated signaling is reduced by GLP-1 signaling via induction of cyclic adenosine monophosphate (cAMP), which activates protein kinase A (PKA). This leads to inhibition of zeta-chain-associated protein kinase 70 (ZAP-70), which lies downstream of TCR activation. This in turn reduces signal transduction through SH2 domain containing leukocyte protein 76 (SLP-76) and resulting gene expression. Induced cAMP also inhibits NF-κB activation, nuclear translocation, and resulting gene expression. cAMP induction leads to increased M2 and decreased M1 macrophage polarization via inhibition of c-Jun N-terminal kinases (JNKs) and thereby transcription factor Jun (cJun)-related gene expression, and via activation of signal transducer and activator of transcription 3 (STAT 3). Dashed lines: indirect pathways. Solid lines: direct pathways. Figures created in BioRender. Povlsen, S. (2025), https://BioRender.com/yjxadfx

There is also accumulating pre-clinical evidence that GLP-1 and GLP-1RAs may modulate several dysregulated inflammatory pathways and immune cell lineages relevant to IBD, although some findings derive from studies in non-intestinal epithelial or immune cell lineages. In rats, liraglutide has been shown to suppress pro-inflammatory macrophage polarization toward the M1 phenotype, accompanied by reduced tumor necrosis factor (TNF)-α gene expression.^53^ In mice, exenatide promoted polarization toward the anti-inflammatory M2 phenotype, associated with tissue repair, via cyclic adenosine monophosphate (cAMP) induction, which activates protein kinase A (PKA), leading to inhibition of c-Jun N-terminal kinase (JNK) phosphorylation and enhanced activation of signal transducer and activator of transcription 3 (STAT3) (Figure 2B).^54–56^ GLP-1 treatment also leads to reduced phosphorylation and nuclear translocation of nuclear factor-κB (NF-κB) in mouse intestinal macrophages (Figure 2B).^57^ NF-κB is a transcription factor central to the regulation of multiple inflammatory genes, including cytokines, chemokines, adhesion molecules, and macrophage polarization markers across various immune cell types.^58–60^ Exenatide may also promote regulatory T cell (Treg) differentiation, a process thought to be impaired in IBD due to disrupted immune tolerance, through increased production of the cytokine transforming growth factor (TGF)-β.^54^^,^^61^

GLP-1R activation in IELs is central to the anti-inflammatory role of GLP-1RAs, where they appear to restrict T cell receptor (TCR) signaling through c-AMP- and PKA-dependent inhibition (Figure 2B).^62^ This leads to decreased pro-inflammatory cytokine production, with *ex vivo* stimulation of IELs with anti-CD3/28 antibodies leading to marked pro-inflammatory cytokine upregulation [IL-2, IL-17A, interferon-γ (IFN-γ), TNF-α], which was significantly attenuated by exenatide.^63^ These findings are supported *in vivo*, where exenatide treatment reduced levels of IL-1β, IL-2, IL-4, IL-5, IL-6, IL-12, TNF-α, IFN-γ, and CXCL1. This effect was abolished in mice lacking GLP-1R expression in T cells, demonstrating a central role for T cell GLP-1R signaling in mediating the anti-inflammatory actions of GLP-1RAs.^62^ GLP-1R expression is seen in both conventional CD4+ and CD8+ T cells expressing TCR αβ, and those expressing TCRγδ (γδ T cells).^63^ γδ T cells are of increasing interest in IBD. Whilst they lack major histocompatibility complex (MHC) restriction and it is unclear which ligands they recognize, they are significantly enriched in the IEL pool in comparison to the periphery and perform critical epithelial barrier surveillance and maintenance functions.^64^^,^^65^ γδ T cell-deficient mice are more susceptible to dextran sodium sulfate (DSS)-induced colitis models, and differential selection of γδ T cells by butyrophilin-like (BTNL) protein polymorphisms has been identified as a risk factor for penetrating Crohn’s disease (CD).^66^^,^^67^ However, the specific lymphocyte subsets responsible for these cytokine effects, particularly the contribution of γδ T cells, remain to be fully elucidated.

GLP-1 has also been shown to regulate colonic expression of angiotensin-converting enzyme (ACE) 2,^68^ higher expression of which has been associated with more severe IBD phenotypes,^69^ and modulation of this axis may benefit patients with IBD. These findings offer a mechanistic foundation for exploring GLP-1RAs as novel anti-inflammatory therapies in IBD, bridging gut physiology with immune modulation.

### 3.2. Gut barrier function

Beyond immune surveillance, epithelial gut barrier function depends on the integrity of the mucin layer—which limits microbial adhesion and invasion—and on tight junctions that maintain epithelial cell–cell adhesion and regulate paracellular permeability to antigens and other substrates.^70^ There is emerging evidence that GLP-1 contributes physiologically to the maintenance of gut barrier integrity. In pre-clinical models, GLP-1 secretion from enteroendocrine L cells is upregulated via toll-like receptor 4 (TLR4) activation in response to increased lipopolysaccharide (LPS) translocation, secondary to impaired barrier function.^71^ The downstream effects of GLP-1R signaling on epithelial integrity are supported by several lines of evidence. In murine models, exenatide administration has been shown to enhance mucin production histologically.^72^ GLP-1R knockout mice exhibit significantly reduced expression of key genes involved in epithelial protection and repair, including trefoil factor (Tff)1, Tff2, TGF-β1, TGF-β3, epidermal growth factor receptor (Egfr), keratinocyte growth factor (Fgf7), and hepatocyte growth factor (Hgf), implicating GLP-1R signaling in mucosal healing pathways.^63^ Moreover, GLP-1RA treatment modulates the expression of additional barrier-associated genes, including IL-33, mucins, and CCL20—although the direction and magnitude of regulation vary depending on the colitis model, disease timepoint, and genetic background, complicating interpretation.^9^ This likely reflects the context-dependent nature of these pathways; IL-33 appears to have important roles in resolution of inflammation and tissue repair, but is also associated with both pro-inflammatory and anti-inflammatory functions in colitis mouse models.^73–75^ Similarly CCL20 appears to have dichotomous roles in intestinal inflammation.^76^^,^^77^

Despite this complexity, liraglutide has been shown to reduce intestinal permeability in LPS-induced gut injury in rats.^78^ Furthermore, GLP-1R signaling appears to have a growth-promoting effect which may enhance its positive effects in gut barrier function, with exenatide increasing small intestinal mass in rats.^79^ This appears to be independent of GLP-2 signaling and occurs through stimulating crypt fission,^80^ and in anti-CD3-treated mouse models of colitis prevents crypt cell death.^62^ These findings support a potential role for GLP-1RAs in preserving epithelial integrity and promoting mucosal repair.

### 3.3. Microbiome

There is clear and consistent evidence of reduced microbial α-diversity in patients with both CD and ulcerative colitis (UC).^81^ The microbiome and immune system engage in bidirectional communication, with dysbiotic microbiota from IBD patients or colitis models shown to exacerbate or induce inflammation when transferred to healthy or susceptible hosts.^82^ The emerging interplay between GLP-1R signaling and the gut microbiota raises the possibility that this pathway may influence microbial composition and host–microbe interactions relevant to IBD.

Comparative studies in GLP-1R knockout and wild-type mice have shown that GLP-1-dependent signaling alters gut microbial composition, with increased abundance of Gammaproteobacteria and Firmicutes, and reduced levels of Actinobacteria and Bacteroidetes.^63^ Further evidence from semaglutide-treated wild-type and IEL-specific GLP-1R knockout mice suggests that IEL-specific GLP-1R signaling modulates the microbiota, increasing *Enterococcus*, *Escherichia–Shigella*, and *Coriobacteriaceae*.^62^ While these compositional shifts do not uniformly align with patterns typically associated with microbiota “healing” in IBD remission,^83^ the absence of colitis models in these studies limits direct extrapolation to therapeutic settings. The complexity of GLP-1R signaling, inflammation, and microbiota crosstalk is further evidenced by the fact that in the gut, germ-free GLP-1R knockout mice exhibit increased mortality and develop colitis phenotypes, both of which can be rescued by transplantation of healthy microbiota.^84^ GLP-1RAs may also affect microbiome composition through slowed intestinal transit, reduced bile acid secretion, and dietary behavioral change in treated patients.^30^ Increased intestinal transit is associated with specific microbiota species,^85^ and patients with bile acid diarrhea effectively treated with colesevalam had a significantly greater abundance of *Fusobacterium* and *Ruminococcus*, both of which help convert primary to secondary bile acids.^86^ Finally, patients taking GLP-1RAs reduce caloric intake by 16%-39%, with some reporting 35%-40% reduction in high-fat food, 12.2% reduction in carbohydrate intake, and significant decreases in cravings for salty, spicy, dairy, and starchy foods.^87^ Although these studies did include microbiota analysis, dietary intake is a major determinate of microbiota composition. Thus, whilst the capacity of GLP-1R signaling to influence the microbiome is evident, direct evidence that GLP-1RAs exert their anti-inflammatory effects in the gut via modulation of the microbiota remains to be established.

Preclinical data demonstrate that GLP-1 and GLP-1RAs exert anti-inflammatory effects, preserve gut barrier integrity, and modulate the microbiome. They attenuate disease severity and histological inflammation in both DSS and T cell-mediated colitis models.^9^^,^^63^ Moreover, GIP also exhibits anti-inflammatory properties in murine models of chemotherapy-induced intestinal injury, suggesting potential additive benefits of dual agonists such as tirzepatide.^88^ As diseases of immune dysregulation, disrupted gut barrier integrity and dysbiosis, GLP-1RAs which target this in IBD are an attractive prospect. There is compelling mechanistic rationale for evaluating GLP-1RAs in clinical studies of IBD, and for further exploring their therapeutic potential beyond metabolic disease.

## 4. GLP-1RA in IBD: clinical evidence

Given the pathophysiological relevance of GLP-1, particularly its immunomodulatory and barrier-enhancing effects, there is growing interest in its potential therapeutic role in IBD. At the same time, concerns persist regarding the gastrointestinal side-effects commonly associated with GLP-1RAs, which may limit their applicability in patients with coexisting IBD. Although no prospective or RCT data are currently available on the use of GLP-1RAs in IBD, the marked rise in their prescription over the past decade has enabled several large retrospective analyses to be conducted.

One of the largest retrospective studies to date, by Villumsen et al.,^89^ utilized Danish national registry data from 2007 to 2019 to assess 3751 patients with IBD (77.5% UC, 22.5% CD) and T2DM. Of these, 982 were treated with a GLP-1RA and/or a dipeptidyl peptidase-4 (DPP-4) inhibitor, while 2769 received other oral antidiabetic agents (Table 1). The primary outcome was a composite of oral corticosteroid use, initiation of TNF-α inhibitors, IBD-related hospitalization, or IBD-related surgery. Among those treated with a GLP-1RA alone (*n* = 469), there was a statistically significant reduction in hospitalization and corticosteroid use, with a numerical reduction in TNF-α inhibitor initiation and surgery. However, the study did not report changes in BMI or HbA1c, both of which may confound IBD outcomes due to known associations between obesity,^90^ diabetes,^91^ and disease severity. Furthermore, most patients (77.8%) had mild disease, limiting extrapolation to more severe phenotypes.

Next, a large Israeli cohort study, evaluated 3737 patients with IBD and T2DM (1883 UC and 1854 CD) over a median follow-up of 6 years.^92^ The primary outcome was a composite of steroid dependency, treatment escalation, IBD-related hospitalization, abdominal or perianal surgery, or death. GLP-1RA use was associated with a reduced risk of adverse outcomes in the full cohort (hazard ratio [HR] 0.79, 95% CI 0.66-0.96), and in UC (HR 0.73, 95% CI 0.54-0.98), though not significantly in CD (HR 0.86, 95% CI 0.66-1.08). While the analysis adjusted for HbA1c, a subgroup analysis showed no benefit in non-obese patients (BMI < 30), again suggesting possible confounding by metabolic improvement. Importantly, neither study incorporated endoscopic assessment, nor did they report on disease location or behavior. Both appear to represent mild disease populations, based on low rates of prior surgery and advanced therapy use (eg, 5.9% advanced therapy use in the Gorelik study), limiting generalizability to the broader IBD population.

**Table 1.** Summary of published studies investigating GLP-1 receptor agonists in inflammatory bowel disease.

Study (author, year, country)	Study type	Population	n	IBD-related outcomes	Findings
**Villumsen et al. (2021, Denmark)**	Retrospective cohort	IBD and T2DM treated with GLP-1RA and/or DPP-4 inhibitor	3751	Composite outcome including need for: Oral corticosteroids TNFα inhibitors IBD-related hospitalization IBD-related surgery	IRR of 0·52 (95% CI: 0·42-0·65) in those treated with GLP-1RA/DPP-4 inhibitors compared to other anti-diabetic medications
**Gorelik et al. (2025, Israel)**	Retrospective cohort	IBD and T2DM treated with GLP-1RA	3737	Composite outcome including: Steroid dependency IBD treatment escalation IBD-related hospitalization or surgery Death	GLP-1RA use was associated with reduced composite outcome in: The full cohort (aHR 0.74, 95% CI 0.62-0.89) Either subtype UC (aHR 0.71, 95% CI 0.52-0.96) and CD (aHR 0.78, 95% CI 0.62-0.99)
**Desai et al. (2024, USA)**	Retrospective cohort	IBD and T2DM treated with GLP-1RA	2270	Composite outcome including: Hospitalization requiring IV steroids IBD-related surgery within 3 years	Lower risk of colectomy (aHR: 0.37, 95% CI: 0.14-0.97) between the UC GLP-1RA and control cohort Lower risk of surgery (aHR: 0.55, 95% CI: 0.36-0.84) between the CD GLP-1RA and CD control cohort. No difference in the risk of intravenous steroid use in UC (aHR: 1.21, 95% CI: 0.92-1.59) or CD (aHR: 1.04, 95% CI: 0.80-1.34) No difference in the risk of oral steroid use or advanced therapy initiation
**Desai et al. (2025, USA)**	Retrospective cohort	IBD patients with obesity treated with GLP-1RA	320	Weight loss and risk of IBD-related complications: need for oral or IV steroids, hospitalization, surgery, treatment escalation	No significant difference in complication rates between GLP-1RA and non-GLP1-RA cohort.
**Levine et al. (2025, USA)**	Retrospective cohort	IBD and GLP-1RA prescription for any indication (T2DM, weight management)	224	Composite outcome including: IBD-related hospitalization Corticosteroid prescription Medication escalation or changes IBD-related surgery	No change in rates of IBD exacerbation, IBD-related hospitalization, steroids prescription, medication escalation or changes, or IBD-related surgery in 12 months after GLP-1RA initiation
**Belinchon et al. (2024, Spain)**	Retrospective case series	IBD & GLP-1RA prescription for weight management	16	IBD activity assessed through HBI or pMayo and FCP Safety profile	No statistically significant change in HBI, pMayo, or FCP after 6 months of therapy Safety profile consistent with previous trial data
**Phan et al (2024, USA)**	Case control	IBD & GLP-1RA (or other anti-obesity medication) prescription for weight management	19	Safety and efficacy of GLP-1RA in IBD assessed through change in symptoms; corticosteroid use, hospitalization or surgery along with change in medication or objective radiological or endoscopic evidence of IBD activity	No difference in side-effects in IBD and non-IBD controls Overall 19.4% experienced flares in 12-month trial period. Rates of flares for each individual medication were not published
**Anderson et al. (2022, USA)**	Retrospective cohort	IBD & GLP-1RA prescription for any indication (T2DM, weight management)	120	Safety and efficacy of GLP-1RA in IBD assessed through: Clinical severity scores (HBI or mMayo) Endoscopic scores (SES-CD or MES) IBD-related hospitalizations Change in CRP	Reduction in CRP 1 year after initiation compared to 1 year prior to GLP-1RA initiation (12.92 vs 6.38 mg/dL, *P *= .005) with correlation between weight and CRP post-GLP-1RA therapy No statistically significant difference in the number of IBD-related hospitalizations, clinical rating scores, or endoscopic scores between the years before and after GLP-1RA initiation
**Nielsen et al. (2024, Denmark)**	Retrospective cohort	IBD & GLP-1RA prescription for any indication (T2DM, weight management)	4430	Risk of ileus or bowel obstruction	No statistically increased risk of ileus associated with GLP-1RA treatment (aHR 0.58; 95% CI 0.34-0.97)
**St Pierre et al. (2024, USA)**	Observational cohort	IBD and GLP-1RA prescription for weight management (non-diabetic population)	36	Tolerability Safety Reduction in CRP	58.3% reported no side-effects 1 patient experience a flare necessitating oral steroids (5 months after GLP-1RA initiation) Toxic thiopurine metabolites in 1 patient having been stable prior to GLP-1RA initiation No significant change in CRP over study period
**Gold et al. (2025, USA)**	Retrospective cohort	IBD & GLP-1RA prescription for any indication (T2DM, weight management)	993	Tolerability Efficacy	Clinical remission (HBI or Mayo) at baseline 74% vs at 3 months (93% *P *< .01), 6 months 81% *P *= .3, 12 months 89% *P = *.09) 23% reported side effects (10% nausea, 9% constipation, 3% diarrhea, 1% injection site reactions).
**Herfarth et al. (2024, USA)**	Placebo-controlled proof-of-concept	Patients with an IPAA and bowel frequency refractory to anti-motility agents with normal pouch morphology	8	Efficacy Safety	Liraglutide reduced bowel frequency by at least 30% in 6/8 (75%) patients compared to 2/8 (25% treated with placebo (*P *= .03) No significant safety concerns identified; nausea seen in 5/8 liraglutide vs 2/8 placebo

Abbreviations: IBD, inflammatory bowel disease; T2DM, type 2 diabetes mellitus; GLP-1RA, glucagon-like peptide-1 receptor agonist; DPP-4, dipeptidyl peptidase-4; TNFα, tumor necrosis factor α; IRR, incidence rate ratio; CI, confidence interval; aHR, adjusted hazard ratio; UC, ulcerative colitis; CD, Crohn’s disease; HBI, Harvey–Bradshaw Index; pMayo, Partial Mayo score; mMayo, modified Mayo; SES-CD, Simple Endoscopic Score-CD; MES, Mayo Endoscopic Score; CRP, C-reactive protein.

A similar observational study from the USA by Desai et al.^93^ used propensity score matching to assess 2270 patients with T2DM and IBD (1130 UC and 1140 CD) over a 3-year period. The study found reduced rates of colectomy in UC (adjusted HR [aHR]: 0.37, 95% CI: 0.14-0.97) and IBD-related surgery in CD (aHR: 0.55, 95% CI: 0.36–0.84) among those treated with GLP-1RAs compared to other oral therapies for diabetes. No differences were observed in intravenous or oral steroid use, or initiation of advanced therapy. However, cohort characteristics again suggest a relatively mild disease population, with only 3.0% of patients having perianal CD, 0.8% with intestinal fistulas, and baseline advanced therapy use in 32% of CD and 19.6% of UC patients in the GLP-1RA group. While subgroup numbers were small, the statistically significant reduction in surgery was observed only with semaglutide, and not with liraglutide or dulaglutide, suggesting that the choice of GLP-1RA may be clinically relevant (Table 2). A subsequent systematic review and meta-analysis found that GLP-1RA use was associated with a decrease in steroid use, reduced need for surgery and fewer hospitalizations, although only nine studies were included and there was significant heterogeneity between the groups included so the results should be interpreted cautiously.^94^

**Table 2.** Currently approved GLP-1 receptor agonists in the UK, EU, and USA.

GLP-1RA	Approval date	Duration	Dosage	Approvals
**Exenatide (Byetta/Bydureon; AstraZeneca)**	2005 (Byetta), 2012 (Bydureon)	Short-acting (Byetta), long-acting (Bydureon)	Byetta: administered twice daily. Bydureon: administered once weekly.	T2DM
**Liraglutide (Victoza/Saxenda; NovoNordisk)**	2010 (Victoza), 2014 (Saxenda)	Short-acting	Victoza: administered once daily. Saxenda: administered once daily.	Victoza: T2DM. Saxenda: obesity and weight management.
**Albiglutide (Tanzeum; GlaxoSmithKline)**	2014	Long-acting	Administered once weekly.	T2DM
**Dulaglutide (Trulicity; Eli Lilly)**	2014	Long-acting	Administered once weekly.	T2DM
**Lixisenatide (Lyxumia/Adlyxin; Sanofi)**	2016	Short-acting	Administered once daily.	T2DM
**Semaglutide (Ozempic/Rybelsus/Wegovy; NovoNordisk)**	2017 (Ozempic), 2019 (Rybelsus), 2021 (Wegovy)	Long-acting	Ozempic: administered once weekly. Rybelsus: administered once daily (oral). Wegovy: administered once weekly.	Ozempic and Rybelsus: T2DM Wegovy: obesity and weight management.
**Tirzepatide—dual GLP-1RA and GIP agonist (Mounjaro/Zepbound; Eli Lilly)**	2022 (Mounjaro), 2024 (Zepbound)	Long-acting	Administered once weekly.	Mounjaro: T2DM Zepbound: obesity and weight management.

Unless otherwise stated, administration is via the subcutaneous route. Abbreviations: GIP, glucose-dependent insulinotropic polypeptide; GLP-1RA, glucagon-like peptide-1 receptor agonists; T2DM, type 2 diabetes mellitus.

While the majority of patients in the aforementioned studies had relatively mild disease phenotypes, a smaller study by Levine et al.^95^ (*n* = 224) included a cohort with more severe disease with 17% of patients affected by perianal involvement and 24% classified as having penetrating or fistulizing disease. In this study, GLP-1RA use was not associated with significant differences in IBD-related hospitalization, steroid use, surgery, or escalation of therapy, although numerical reductions in all these outcomes were observed in the GLP-1RA-treated group. Additionally, a small retrospective series described 17 patients with perianal fistulizing CD treated with a GLP-1RA, reporting improvements in fistula drainage and pain scores.^96^ However, this study lacked a control group and did not assess healing radiologically or endoscopically.

Data from a proof-of-concept study also found that, in patients with an ileoanal pouch anastomosis and bowel frequency refractory to anti-motility agents despite normal pouch morphology, liraglutide significant reduced bowel frequency compared to placebo (*P *< .03).

A smaller number of studies have attempted to assess the therapeutic impact of GLP-1RA on objective disease activity using biomarkers and endoscopic scores. The previously described study by Desai et al.^93^ also included data on fecal calprotectin (FCP), showing numerically lower levels in both UC and CD among GLP-1RA-treated patients, though the differences were not statistically significant. A retrospective study from Spain assessed FCP and clinical indices (partial Mayo for UC and Harvey–Bradshaw Index [HBI] for CD) in 16 patients with IBD prescribed GLP-1RA for obesity (five with semaglutide, 11 with liraglutide).^97^ No significant changes in FCP or clinical scores were observed, although baseline disease activity was low (mean FCP 42 µg/g, HBI 3, and partial Mayo 1.3). A further study presented at Digestive Disease Week 2025 of 993 patients found GLP-1RA to be associated with a statistically significant increase in clinical remission rates (HBI or Mayo score) at 3 months numerical at 6 and 12 months without an increase in side-effects.^98^

A single-center study from the USA evaluated 120 patients with IBD, of whom 60.8% were on an advanced therapy at baseline, and reported numerical reductions in HBI (3.52–3.18), modified Mayo score (1.61–1.54), and Simple Endoscopic Score for CD (SES-CD) (3.1–2.6) 1 year after GLP-1RA initiation.^99^ Notably, a statistically significant reduction in C-reactive protein (CRP) was observed (12.92–6.38 mg/L, *P *= .005), a finding echoed in a separate study of 222 patients with CD treated with GLP-1RA, though in UC this effect was only numerical.^100^ This study also reported a significant decrease in FCP in CD, but a numerical increase in UC.^100^ However, the investigators excluded patients who required steroids or experienced treatment escalation, potentially introducing bias by omitting those who flared. Finally, a small study of 36 non-diabetic IBD patients treated with semaglutide or tirzepatide found no significant change in CRP and had insufficient data to evaluate FCP.

### 4.1. Are improved outcomes related to weight loss or a direct effect of a GLP-1RA?

Taken together, the available data suggest that GLP-1RA use in patients with IBD may be associated with improved outcomes. However, both visceral and subcutaneous fat play significant but differing roles in systemic inflammation^101^^,^^102^ and it is therefore possible that the improved outcomes are the result of improvements in obesity and T2DM rather than a direct effect of the GLP-1RA. This hypothesis was supported by the Gorelik study which found no benefit in non-obese patients^92^ whilst a smaller study by Anderson et al. found no correlation between CRP reduction and weight loss,^99^ leaving the question unresolved. Regarding disease activity, the evidence—based on clinical, biochemical, and endoscopic markers—suggests a potential benefit, but remains inconclusive. There are also data that suggest that bile acid malabsorption can be successfully treated with GLP-1RA^103^ and this may be an important confounder in registry studies that was not corrected for. Large prospective studies are needed to determine causality and clinical relevance.

### 4.2. Metabolic risk in patients with IBD

While evidence remains mixed regarding the direct impact of obesity on IBD disease activity, the literature consistently demonstrates that obesity is associated with an increased risk of surgical complications,^104^ particularly post-operative infections where a systematic review found an odds ratio of 1.48 compared to non-obese patients with IBD, technical challenges in the creation of an ileal pouch–anal anastomosis or stoma^105^ and parastomal hernia development.^106^ Obesity and other components of the metabolic syndrome are well-established risk factors for cardiovascular and cerebrovascular events. Importantly, IBD itself has also been independently linked to a heightened risk of ischemic heart disease, peripheral arterial disease, acute arterial events, and stroke.^107–109^ It is therefore reasonable to hypothesize that weight reduction and improvements in metabolic parameters may lower cardiovascular risk in patients with IBD, representing a potential additional benefit of GLP-1RA therapy. Hepatic steatosis, a further component of the metabolic syndrome, is known to be more common in patients with IBD^110^ and GLP-1RAs have previously been shown to be effective for reducing liver fat content,^111^ and this represents another potential benefit to GLP-1RA therapy.

### 4.3. Changes in body composition

Sarcopenia is common in IBD with up to 52% of those with CD and 37% of those with UC meeting the criteria in a systematic review.^112^ It is also associated with a negative impact on IBD outcomes including post-operative complications, length of hospital stay, and the need for surgery.^113–115^ GLP-1RAs, which are associated with a reduction in lean muscle mass as well as adipose tissue loss,^116^ may therefore put patients with IBD at a greater risk of sarcopenia, and prospective data are needed to further investigate.

## 5. Clinical implications for patients with IBD

Beyond their potential impact on disease activity, GLP-1RAs carry important implications for the broader care of patients with IBD including effects on metabolic health, psychosocial wellbeing, and considerations around adverse effects and safety. While further research is needed to define the efficacy and safety of GLP-1RAs specifically in IBD, these agents are already widely prescribed for comorbid obesity and T2DM. Moreover, increasing numbers of patients are independently seeking out these therapies—often outside formal medical pathways—to self-manage perceived obesity. This expanding and sometimes unsupervised use of GLP-1RAs raises immediate and practical considerations for clinical management in patients with IBD.

### 5.1. Safety of GLP-1RA in IBD

The most robust and contemporary data show that in the general population, GLP-1RAs are generally well tolerated and have a favorable safety profile.^117^ However, the gastrointestinal side-effect profile of GLP-1RAs has raised concerns regarding their use in patients with IBD, particularly due to the potential for symptom exacerbation.

Whilst diarrhea occurs in up to 20% of patients treated with GLP-1RA,^118^ national registry studies from Denmark,^89^ Israel,^92^ and the USA,^93^ collectively involving over 10 000 patients with IBD, found no indication that GLP-1RA use was associated with worsening of IBD symptoms or increased adverse event rates. These findings are further supported by smaller studies involving a variety of GLP-1RAs, which similarly reported no increase in IBD-related complications or gastrointestinal adverse events (AEs).^95^^,^^97^^,^^99^^,^^119^ Taken together, current data suggest that the risk of gastrointestinal AEs in patients with IBD treated with GLP-1RAs does not exceed that observed in the general population.

Nausea and vomiting are common side-effects of GLP-1RAs, affecting 15%-59% and 5%-20% of patients respectively^120^ and are thought to result from delayed gastric transit. Non-pharmacological treatment strategies such as dietary modifications for gastroparesis^121^ are appropriate in the first instance, with prokinetics such as domperidone or prucalopride used in refractory cases. Since satiety and reduced appetite are key contributors to weight loss, managing these symptoms while maintaining the therapeutic efficacy of GLP-1RAs should be carefully balanced.^122^

Gastro-esophageal reflux disease (GORD) is more prevalent in obese patients; however, a large retrospective cohort study by Liu et al. reported an 11% increased risk of GORD in individuals treated with short-acting GLP-1RAs.^123^ This association was not observed with long-acting GLP-1RAs. Standard management of GORD is recommended, with consideration given to dose reduction or discontinuation of the GLP-1RA if symptoms persist despite initial interventions.^124^

Whilst there have been suggestions of a potential association with intestinal obstruction,^125^ a Danish health registry analysis of 4430 patients with IBD found no increased risk of bowel obstruction or ileus associated with GLP-1RA.^126^ Early clinical trials and real-world use raised concerns about an increased risk of acute pancreatitis,^127^ although more recent data from a propensity score-matched analysis in almost 100 000 patients using GLP-1RA have not confirmed this association;^128^ caution is still recommended in patients with predisposing risk factors or a history of pancreatitis. A systematic review and meta-analysis encompassing 76 RCTs and over 100 000 patients found that GLP-1RA use was associated with an elevated risk of gallbladder and biliary disease, such as cholelithiasis (relative risk [RR] 1.27), cholecystitis (RR 1.36) and other biliary diseases including biliary colic, biliary cysts, cholangitis, and biliary duct obstruction (RR 1.55).^129^ These risks were greater with higher doses and longer durations of use.^129^

Data have also repeatedly demonstrated an increased risk of thyroid cancer (aHR 1.58 for all types at 3 years; 1.78 in medullary thyroid cancer).^130^ Earlier GLP-1RAs such as exenatide and liraglutide were linked to acute kidney injury^131^ with case reports of acute interstitial nephritis (AIN).^131^ There are conflicting data with regard to the more novel agents, with case reports linking semaglutide to AIN^132^ whilst a meta-analysis published in 2024 showed that GLP-1RAs, including semaglutide, are associated with a decreased risk of kidney injury (odds ratio 0.84, *P *< .001) regardless of baseline renal function.^133^

### 5.2. Psychosocial health

Psychological comorbidity is common in IBD,^134^ and in a study of 7000 participants with IBD rates of depression, fatigue, anxiety, and pain were greater in those who were obese.^14^ Obese individuals also demonstrated a greater impairment in social function and this difference persisted even when accounting for disease activity.^14^ Similarly, diabetes was found to be associated with poorer quality of life in those with IBD.^135^ It is therefore possible that the positive impact of GLP-1RA on obesity and glycemic control may confer psychosocial benefits to patients with IBD. However, there are also data suggesting that GLP-1RA use may be associated with worsening mental health disorders,^136^ discussed in detail below, and further longitudinal studies are required.

### 5.3. Practical prescribing considerations in IBD

In line with regulatory guidance, GLP-1RAs are contraindicated in patients with end-stage renal impairment, or those with an estimated glomerular filtration rate < 30 mL/min when using exenatide. Other contraindications include a history of pancreatitis, severe hepatic impairment, and severe gastrointestinal disease.^137^ Use is also contraindicated in individuals with a personal or family history of medullary thyroid carcinoma or multiple endocrine neoplasia.^138^ Caution is additionally advised in patients with heart failure, retinopathy, and women of childbearing potential. GLP-1RA use has also been shown to reduce the absorption of the oral contraceptive pill, warranting appropriate counseling and consideration of alternative contraceptive methods prior to initiation.^139^

Although generally well tolerated, meta-analysis of 14 clinical trials suggests that long-acting GLP-1RAs (eg, semaglutide, tirzepatide) are associated with lower rates of gastrointestinal side-effects, particularly nausea and vomiting, compared to short-acting agents (Table 2).^140^ However, diarrhea may be more pronounced with longer-acting formulations, and these differences should be factored into treatment selection and individualized care plans.

GLP-1RA use is known to delay gastric emptying,^141^ contributing to nausea in some patients.^142^ This has important implications for individuals with IBD, as delayed gastric transit may impair the absorption of orally administered therapies such as 5-ASA and thiopurines. A case report showed a three-fold increase in 6-methylmercaptopurine and two-fold increase in 6-thioguanine levels with associated new derangement in liver function tests indicating toxicity following the initiation of tirzepatide.^143^ Obesity can also significantly influence the pharmacokinetic profile of therapeutics used in IBD. Increased volume of distribution, particularly in individuals with higher adipose tissue mass and elevated expression of Kruppel-like factor 4, may alter drug metabolism and clearance rates.^144^ This can pose challenges in achieving therapeutic serum concentrations of agents such as anti-TNF therapies^17^ and thioguanine metabolites.^145^ In patients undergoing weight loss with GLP-1RA therapy, clinicians should therefore remain vigilant to the potential for substantial shifts in drug pharmacokinetics. Monitoring and dose adjustment of advanced therapies may be necessary to ensure therapeutic efficacy and avoid AEs as body weight and distribution change.

The altered gastrointestinal transit seen with GLP-1RAs has also been shown to negatively impact the efficacy of bowel preparation regimes in ileocolonoscopy^146^ and is associated with reduced small bowel visualization with video capsule endoscopy.^147^ The risk of aspiration, due to retained gastric contents, is also a concern when performing upper gastrointestinal endoscopy. Holding GLP-1RAs prior to procedures has previously been advised, but this can negatively impact glycemic control and it is unknown if withholding one dose is sufficient to allow gastrointestinal transit to return to normal. Guidance from the American Gastroenterology Association published in 2024^148^ advises that in patients without symptoms of gastroparesis, standard pre-procedural fasting is safe and appropriate. However, in patients with symptoms suggestive of gastroparesis, a bedside ultrasound to assess for a stomach volume of <150 mL or a liquid diet for the day preceding the procedure are advised instead of holding GLP-1RAs pre-procedurally.

Gallbladder and biliary disease are common findings in patients with IBD^149^ and given the known risks from GLP-1RA as described above, patient education regarding important symptoms to be aware of is crucial when initiating these treatments and caution is advised in patients with a history of biliary disease.^124^

Many mental health disorders, particular depression and anxiety, are more common in IBD.^150^ A large cohort study included 162 253 patients taking liraglutide or semaglutide and found a 195% increased risk of major depression and a 108% increased risk of anxiety compared to controls.^136^ Whilst there are no data in IBD, given the higher prevalence of mental health disease in the IBD population, screening prior to treatment initiation and consideration of enhanced monitoring is advised.

### 5.4. Current recommendations for patients with IBD

**Figure 3.**
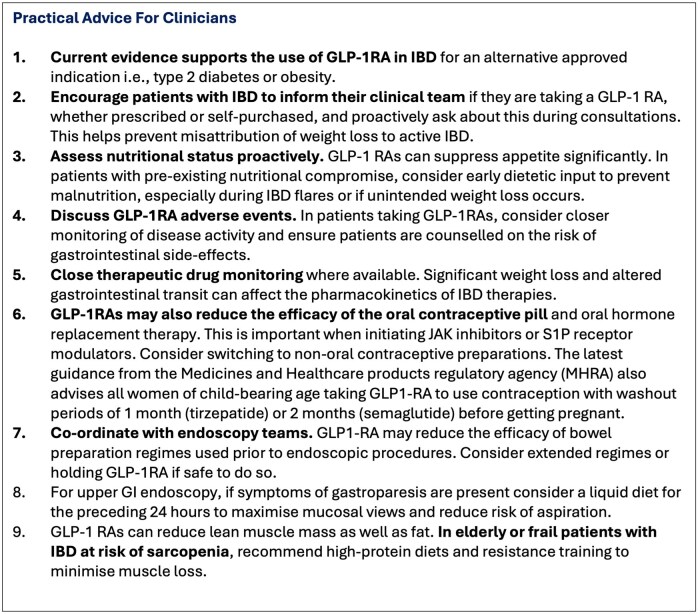
Practical advice to clinicians when managing patients with IBD who are taking GLP-1RAs.^112^^,^^155^

Current evidence supports the use of GLP-1RAs in IBD patients who require the medication for an alternative approved indication, provided there is appropriate monitoring for potential side-effects. Access to GLP-1RAs should not be denied to individuals with approved co-existing indications, given the established benefits in these contexts. Practical considerations are summarized in Figure 3.

At present, there is no evidence to support the use of GLP-1RAs as primary therapy or as adjunctive treatment alongside other advanced therapies specifically for IBD or for IBD patients with metabolic risk factors. Future research should prioritize investigating the dual benefits of metabolic control and potential inflammatory modulation. Candidate patient groups for such dual-treatment approaches may include IBD patients with elevated BMI, those at increased risk of metabolic complications, patients receiving therapies associated with heightened cardiovascular risk, and overweight individuals with IBD who are candidates for surgical intervention.

## 6. Future directions and conclusions

Well-designed prospective trials are essential to establish whether GLP-1RAs have a definitive impact on IBD disease course, activity, and safety in affected populations. Such studies should incorporate objective measures of disease activity, including endoscopic assessment, and include representative patient cohorts.

Ongoing clinical trials are planned, including evaluating tirzepatide as adjunctive therapy in individuals with a BMI over 27 who are already receiving infliximab or adalimumab treatment (NCT06774079).^115^ This study also includes a dietary counseling arm and aims to assess not only clinical outcomes, but also changes in inflammatory biomarkers and quality of life measures. The results of this trial may provide valuable insight into whether GLP-1RA-mediated weight loss can enhance therapeutic response in CD.^151^ Two multicenter, double-blind, placebo-controlled randomized trials (NCT06937086 and NCT06937099) designed to assess the efficacy and safety of tirzepatide in patients with UC and CD respectively are due to start imminently.^152^^,^^153^ The primary objective of these studies is to determine whether combining mirikizumab with tirzepatide, as opposed to mirikizumab with placebo, results in a reduction or resolution of symptoms and achieves a weight loss of at least 10% of total body weight. These trials will also provide much-needed prospective data on the safety of GLP-1RA in patients with IBD.

Future studies are also required to assess the indirect impact of GLP-1RA on dietary habits and how this may affect disease activity. It is known that reducing pro-inflammatory food items from a patient’s diet can improve outcomes in IBD^154^ but it remains unclear if, either unintentionally through centrally mediated appetite suppression or intentionally as part of a conscious move towards improved health, GLP-1RA use may result in dietary changes that reduce intestinal inflammation and improve IBD outcomes.

GLP-1RAs hold promise as dual-action agents in IBD, offering potential benefits in metabolic control, weight loss, and disease modulation through immunological and barrier-enhancing effects. Pre-clinical findings and early retrospective data are encouraging; however, current evidence remains insufficient to draw firm conclusions, especially concerning mechanistic impacts on disease activity, optimal timing of GLP-1RA initiation, safety considerations in the IBD population and potential pharmacological interactions. Ongoing prospective trials incorporating objective endpoints and representative IBD populations are essential. In the meantime, given the widespread and often unsupervised use of GLP-1RAs, clinicians must individualize care, carefully weighing potential benefits against the complex safety considerations in this patient group.

## Data Availability

No new data were generated or analysed in support of this research.
